# The implementation of NILS: A web-based artificial neural network decision support tool for noninvasive lymph node staging in breast cancer

**DOI:** 10.3389/fonc.2023.1102254

**Published:** 2023-03-01

**Authors:** Looket Dihge, Pär-Ola Bendahl, Ida Skarping, Malin Hjärtström, Mattias Ohlsson, Lisa Rydén

**Affiliations:** ^1^ Department of Clinical Sciences Lund, Division of Surgery, Lund University, Lund, Sweden; ^2^ Department of Plastic and Reconstructive Surgery, Skåne University Hospital, Malmö, Sweden; ^3^ Department of Clinical Sciences Lund, Division of Oncology, Lund University, Lund, Sweden; ^4^ Department of Clinical Physiology and Nuclear Medicine, Skåne University Hospital, Lund, Sweden; ^5^ Department of Astronomy and Theoretical Physics, Division of Computational Biology and Biological Physics, Lund University, Lund, Sweden; ^6^ Department of Surgery, Skåne University Hospital, Malmö, Sweden

**Keywords:** breast cancer, artificial intelligence, neural networks, disease diagnosis, lymphatic metastasis, sentinel lymph node, clinical decision support, programmable calculator

## Abstract

**Objective:**

To implement artificial neural network (ANN) algorithms for noninvasive lymph node staging (NILS) to a decision support tool and facilitate the option to omit surgical axillary staging in breast cancer patients with low-risk of nodal metastasis.

**Methods:**

The NILS tool is a further development of an ANN prototype for the prediction of nodal status. Training and internal validation of the original algorithm included 15 clinical and tumor-related variables from a consecutive cohort of 800 breast cancer cases. The updated NILS tool included 10 top-ranked input variables from the original prototype. A workflow with four ANN pathways was additionally developed to allow different combinations of missing preoperative input values. Predictive performances were assessed by area under the receiver operating characteristics curves (AUC) and sensitivity/specificity values at defined cut-points. Clinical utility was presented by estimating possible sentinel lymph node biopsy (SLNB) reduction rates. The principles of user-centered design were applied to develop an interactive web-interface to predict the patient’s probability of healthy lymph nodes. A technical validation of the interface was performed using data from 100 test patients selected to cover all combinations of missing histopathological input values.

**Results:**

ANN algorithms for the prediction of nodal status have been implemented into the web-based NILS tool for personalized, noninvasive nodal staging in breast cancer. The estimated probability of healthy lymph nodes using the interface showed a complete concordance with estimations from the reference algorithm except in two cases that had been wrongly included (ineligible for the technical validation). NILS predictive performance to distinguish node-negative from node-positive disease, also with missing values, displayed AUC ranged from 0.718 (95% CI, 0.687-0.748) to 0.735 (95% CI, 0.704-0.764), with good calibration. Sensitivity 90% and specificity 34% were demonstrated. The potential to abstain from axillary surgery was observed in 26% of patients using the NILS tool, acknowledging a false negative rate of 10%, which is clinically accepted for the standard SLNB technique.

**Conclusions:**

The implementation of NILS into a web-interface are expected to provide the health care with decision support and facilitate preoperative identification of patients who could be good candidates to avoid unnecessary surgical axillary staging.

## 1 Introduction

Breast cancer in females is the most commonly diagnosed cancer, with approximately 2.3 million new annual cases worldwide (1). Breast cancer care has evolved into a multidisciplinary approach, with increasingly conservative surgical techniques in the breast and axilla and targeted systemic therapies (2). Evaluation of axillary nodal status in primary breast cancer determines the pathological stage of the disease, and the presence of axillary lymph node metastasis remains a key prognostic factor in breast cancer (3). Therefore, an accurate assessment of nodal status is still crucial in guiding the extent of axillary surgery and the need for neoadjuvant and adjuvant therapy including postoperative radiotherapy. Over the decades, several landmark trials have established a more conservative and less extensive approach for axillary nodal staging, from axillary nodal dissection (ALND) to today’s standard staging by sentinel lymph node biopsy (SLNB) (4–6). Furthermore, previous routine completion of axillary lymph node excision in sentinel lymph node-positive breast cancer has been abandoned by the results of randomized trials on axillary management (7–10) and further advances in adjuvant treatment. While paradigm-changing studies such as the ACOSOG Z0011 study (10) indicate an increasing interest in limiting axillary surgery, and early detection by mammography screening has shifted the distribution of breast cancer toward earlier stages (11), SLNB is still routinely performed in patients with clinically node-negative breast cancer. The vast majority of these patients present with benign axillary lymph node status, and the invasive surgical procedure has no therapeutic benefit but increases the risk of morbidity related to pain, seroma, neuropathy, arm lymphedema, and postoperative infection (12–16). In 2021, ASCO published an updated guideline on axillary staging which included recommendations to forego SLNB in selected patients and endorsed a case-by-case evaluation to warrant patient-centered axillary treatment (17).

Although imaging methods for assessing the lymph nodes of the axilla have shown promising results (18–20), the diagnostic accuracy of imaging techniques alone has been insufficient to replace SLNB for nodal staging. Therefore, alternative methods for surgical staging of axillary lymph nodes have been presented, for example, prediction models that combine clinical and tumor biological and/or radiological properties to estimate the probability of axillary lymph node metastasis. Machine learning-assisted models, including artificial neural networks (ANN), have been proposed to supplement standard statistical methods in classification tasks and decision support in cancer (21). An ANN model can handle non-predefined relations, such as nonlinear interactions, and does not require distributional assumptions. However, this technique comes at the cost of reduced interpretability and difficulty in gaining insight into the model features (22).

In 2019, our research group presented an ANN prototype for preoperative noninvasive lymph node staging (23). This original model utilized fifteen patient-related and clinicopathological characteristics to predict healthy lymph nodes and showed better discriminatory ability than a multivariable logistic regression model. The fifteen variables in the model were selected based on association to nodal status in previously published models, variable selection in the ANN prototype and their availability in routine breast cancer work-up. The potential to abstain from surgical axillary staging by SLNB was shown in 18−27% of newly diagnosed breast cancer patients. A health-economic decision-analytic model demonstrated that the implementation of the ANN prototype is associated with substantial cost reductions and overall health gains (24).

This study aims (1) to present the implementation of a further improved ANN algorithm for nodal status prediction in early breast cancer into a web-based tool for noninvasive lymph node **s**taging (NILS), (2) to report on the predictive performance of the NILS tool with integrated ANN models to handle different access to preoperative data, (3) to assess the clinical utility by estimating possible SLNB reduction rates applying the NILS tool to predict nodal status in early breast cancer.

## 2 Materials and methods

### 2.1 Data collection for model development

The current NILS tool is a further development of an ANN prototype for the prediction of healthy lymph nodes published in 2019 (23). The study population for training and internal validation of the initial ANN algorithm was based on a prospectively maintained pathological database. We identified consecutive clinically node negative patients with invasive breast cancer who underwent primary breast surgery and surgical axillary lymph node staging between January 1, 2009, and December 31, 2012, at Skåne University Hospital, Lund, Sweden. Ethical permission was obtained from the local ethics committee at Lund University (LU 2012/340), and patients were allowed to opt out of the study. Exclusion criteria were: male gender, prior ipsilateral surgery in the breast or axilla, palpable axillary lymphadenopathy, neoadjuvant therapy, and unknown pathological nodal status after routine surgical axillary staging. Fifteen potential clinicopathological predictors of nodal metastases were identified. Information on the mode of breast cancer detection (detected at screening mammography vs. symptomatic presentation) was obtained from the mammography screening records. Histological classification and definition of axillary nodal status has previously been described (23). The following data were collected from medical records and surgical pathology reports: age at diagnosis, menopausal status, body mass index, bilateral disease, tumor localization in the breast, tumor multifocality, tumor size, histological type, histological grade, status of estrogen receptor (ER), progesterone receptor (PR), human epidermal growth factor receptor 2 (HER2), Ki67 value, and presence of vascular invasion (VI).

### 2.2 The ANN structure

#### 2.2.1 Variable selection

The initial ANN algorithm for predicting healthy lymph nodes vs. metastatic lymph nodes (N0 vs. N+) previously reported in 2019 required multiple preoperative and postoperative clinicopathological inputs variables (23). Missing data were handled using multiple random imputations. The selection of variables from this first predictive ANN model provided a set of candidate predictors for the current NILS tool. The key features of the variable selection step are summarized here. Ranking of the input variables was performed by determining the input weights of the candidate predictors, and variable redundancy reduction was obtained by maintaining only the top-ranked variables for the prediction of healthy lymph nodes. The overall significance of the input variables was evaluated using a permutation technique. More specifically, a given input variable was decoupled from the patient by a random permutation across the evaluation cohort and the impact of this randomization on predictive ability was assessed. The predictor variable that is associated with the greatest reduction in predictive ability was assigned an importance value of one. All other predictors were given positions according to the associated decrease in the predictive performance at randomization. The 10 highest-ranking variables provided the contribution profile of the final input variables to the current NILS tool. The current tool included in order of importance: largest tumor size, VI, tumor multifocality, ER status, histological type, PR status, mode of detection, age at diagnosis, tumor localization, and Ki67 value.

#### 2.2.2 Multilayer perceptron model

Ensemble models, each comprising 30 multilayer perceptron (MLP) neural networks, were used for the development and internal validation of predictive ANN models. An overview of the original ANN structure has been presented in a previous publication (23). For completeness, we present the model training and internal validation steps in detail.

Each MLP in an ensemble had one hidden layer with 10 nodes and a single output node encoding the probability of healthy axillary lymph nodes. The number of input nodes was 12−16, depending on the specific combination of input features. The activation function for the hidden nodes is a hyperbolic tangent function and a logistic function for the output node. An ensemble prediction was performed by averaging the individual MLP predictions.

#### 2.2.3 Workflow for N0 prediction

**Figure 1:**
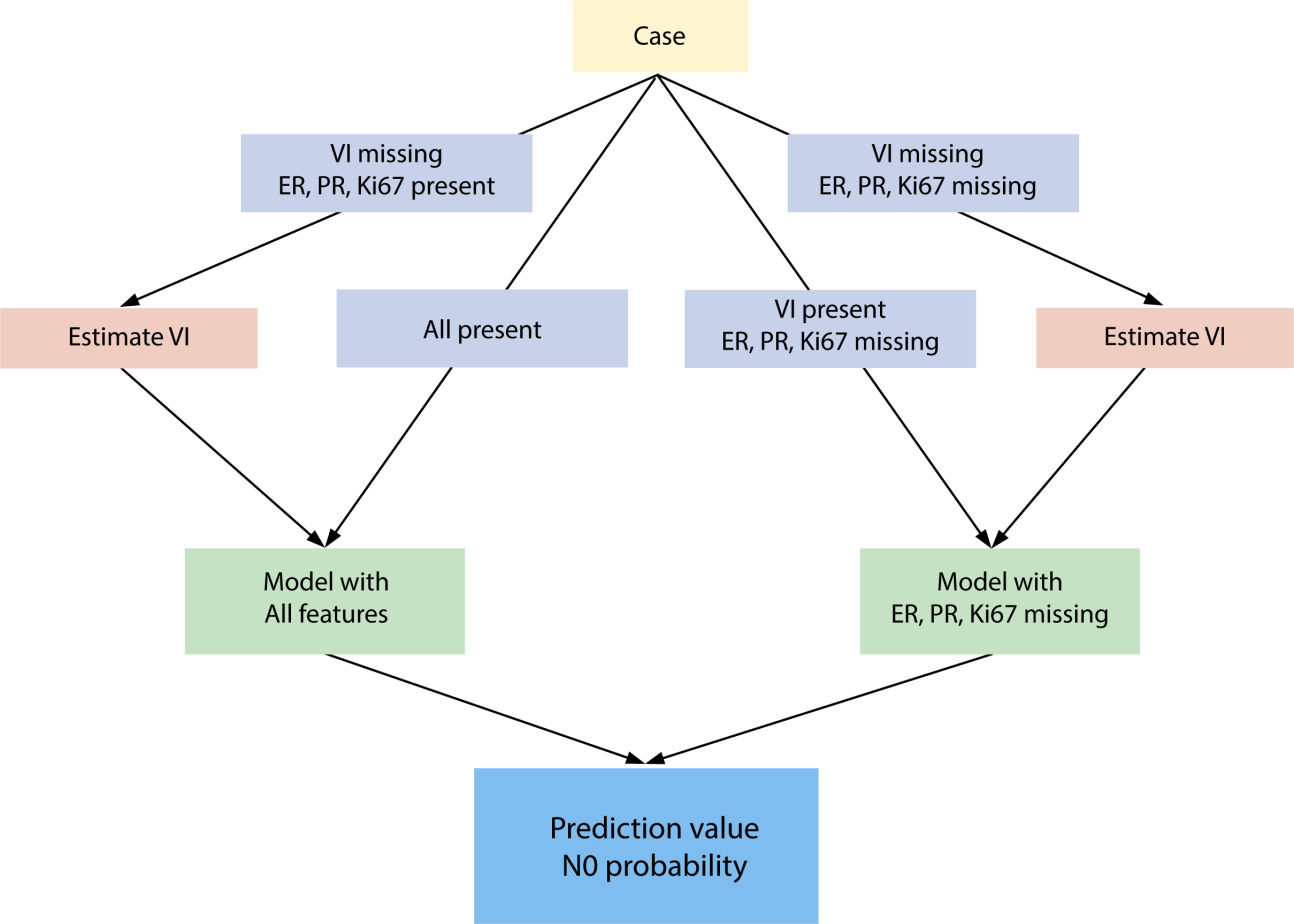
Artificial neural network workflow to estimate the probability of healthy lymph nodes. ER, estrogen receptor; PR, progesterone receptor; VI, vascular invasion; N0, no axillary lymph node involvement (benign lymph nodes).

The present NILS ANN algorithm was trained to handle combinations of missing data for 10 input variables. Strategies for handling missing input variables have been developed to reflect the clinical pathway of diagnostic workup in breast cancer, with a special focus on the risk of incomplete preoperative histopathological reporting. Therefore, the prediction model required that all variables should be present, except for VI and/or ER status, PR status, and Ki67 value. **Figure 1** shows the N0 prediction workflow, which allows combinations of variable inputs.

In the first step, the application programming interface (API) verifies whether information on VI is missing. In cases with missing VI, one of the two imputation models was chosen to acquire an imputed value for VI. The choice of the VI imputation model was based on the presence of ER status, PR status, and Ki67 value. If any of these three input variables were missing, a second model without these inputs was applied. In the next step of N0 prediction after the inclusion of VI, one of the two models was selected in the same manner as described above. If any of the three input variables (ER status, PR status, and Ki67 value) were missing, a model without these features was applied; otherwise, an algorithm using all values was assigned.

#### 2.2.4 Model training

For each model presented in the N0 prediction workflow (**Figure 1**), an ensemble of MLPs was trained. Each MLP was trained on a unique subsample of the original training dataset. Such a sample was created by first randomly splitting the original training dataset into three parts of equal size stratified by class. One part was removed from the data, leaving two parts that formed a training dataset. This procedure was repeated 10 times with different random splits into three parts. In total, 10 × 3 training datasets were created and used to train 30 MLPs in an ensemble.

#### 2.2.5 Model selection

**Figure 2:**
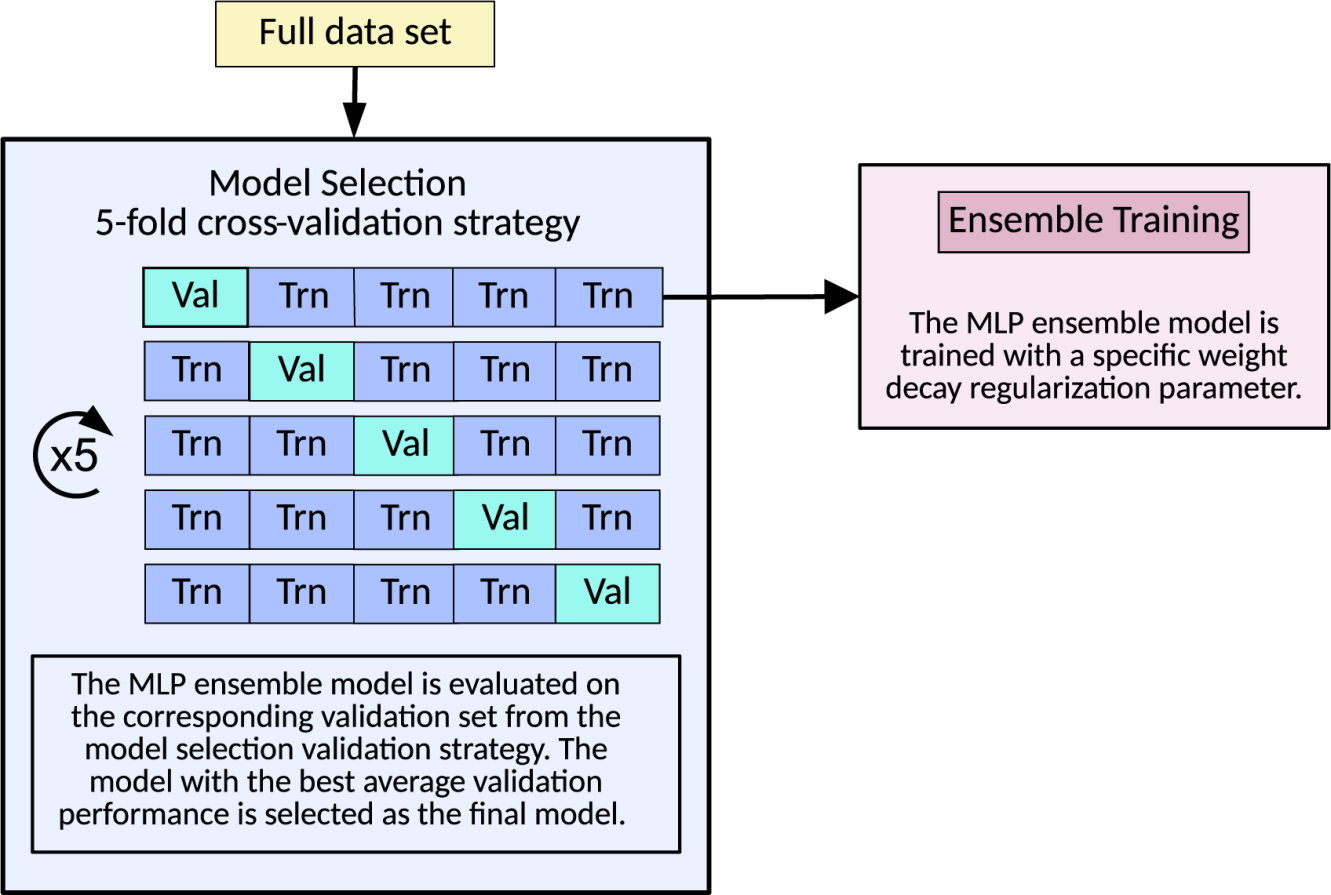
Overview of the model selection procedure based on repeated 5-fold cross-validation. Val, validation; Trn, training; MLP, multilayer perceptron.

Each individual MLP was regularized using the weight decay method (25). This method uses a hyperparameter that sets the amount of regularization to be used. The same weight decay parameter was used for all MLPs in the ensemble, and the optimal parameters were determined using a model selection procedure. Model selection was accomplished using the method of repeated 5-fold cross-validation. The original training dataset was split randomly into five parts of equal size, with a preserved class fraction within each part. Each part was used to validate an ensemble model trained on the remaining four parts. This procedure was repeated five times, resulting in 5 × 5 validation results. The average of these 5 × 5 results was used to validate the performance of the ensemble model. This repeated 5-fold cross-validation procedure was performed for a sequence of different weight decay parameters. The weight decay parameter corresponding to the best 5-fold cross-validation performance was selected as the optimal parameter and was used in the final ensemble model. An overview of the model selection process is shown in **Figure 2**.

#### 2.2.6 Data preprocessing

A maximum of 10 input variables contributed to 16 values used as inputs for the MLP according to the following criteria

1) Tumor size (mm): single numerical value. Normalized to unit standard deviation and zero mean before training.
2) VI: a single binary value. Present = 1, Absent = 0
3) Multifocality: Single binary value. Present = 1, Absent = 0
4) ER status: Single binary value. Positive = 1, Negative = 0
5) Histological type: categorical, three values. Invasive ductal cancer of no special type (NST) (1,0,0), invasive lobular cancer (ILC) (0,1,0), and histological subtypes other than pure NST or ILC (0,0,1).
6) PR status: Single binary value. Positive = 1, Negative = 0
7) Mode of detection: Single binary value. Mammographic screening = 1; symptomatic presentation = 0
8) Age (years): Single numerical value. Normalized to unit standard deviation and zero mean before training.
9) Tumor localization: semi-categorical, five values. Breast tumor localization was computed using two features, the right/left specification and the o’clock position of the tumor, and assigned as central or within the breast quadrants: upper inner (UIQ), upper outer (UOP), lower inner (LIQ), and lower outer (LOQ).
10) Ki67: Single numerical value. Normalized to unit standard deviation and zero mean before training.

### 2.3 Reporting on model predictive performance, calibration, and statistical analysis

The development of the NILS predictive tool for healthy lymph nodes and the report of findings were following an EQUATOR Guideline for reporting machine learning predictive models (26). The predictive performance of the model was represented by the area under the receiver operating characteristics curves (AUC). Sensitivity and specificity values at defined cut-off point were also presented. The reported performances of the different ANN models were obtained from the internal 5-fold procedure. No separate test set or additional cross-validation loop was used for more unbiased estimation of prediction performance. To further simplify the reporting of the performance measures, a single validation result was computed from the internal 5 × 5 fold. Each of the five validation lists was fused to a single validation list, incorporating all training data. Repeated cross-validation led to five validation lists, and a single final list was computed by averaging the predictions for each patient. The reported performance measures (e.g. AUC) were computed from the final validation list and the Hosmer-Lemeshow (HL) chi-squared statistic was used to assess the calibration. Predictive and calibration performances were evaluated using different ANN models within the current NILS tool.

The true positive (TP), true negative (TN), false positive (FP), and false negative (FN) outcomes were evaluated. The false negative rate (FNR) was computed as the number of FN cases divided by the number of cases with confirmed lymph node metastasis FNR= FN/(FN+TP). Possible SLNB reduction rate, defined as (TN + FN)/(TN + FN + TP + FP), was evaluated at a cut-off point equivalent to the maximum FNR of 10%, to reflect the FNR associated with the SLNB technique. Sensitivity TP/(TP + FN) and specificity TN/(TN+FP) values were assessed at the same cut-off point.

The distribution of clinicopathological variables and mode of detection across the nodal status outputs (comparison between N0 and N+) was assessed using Pearson’s χ^2^ test for categorical variables and Mann–Whitney U test for variables measured on a continuous scale. Custom-made software written in C (GCC) version 7.5.0, Perl version 5.26.1, SPSS Statistics for Windows version 25.0 (IBM, Armonk, New York, USA), and Stata version 17 (StataCorp, College Station, Texas, USA) were used for statistical calculations and graphics. A *P*-value should not be interpreted in relation to a cut-off point for significance, but as a continuous measures of evidence against the corresponding null hypothesis.

### 2.4 The benchmark criteria for N0 status reflect the accuracy of the SLNB technique

A cut-off point reflecting the accepted FNR of the SLNB procedure was selected to predict N0. This level of cut-off point, corresponding to 10% FNR, was intended to discriminate N0 vs. N+ and identify individuals with a low probability of axillary metastatic disease, where the omission of surgical axillary staging by SLNB would be supported by the NILS tool. Two cut-off point values were applied: one for the model with all input variables present and one for the model with missing input variables (ER status, PR status, and Ki67 value).

### 2.5 Development of a user-friendly interface for personalized prediction of N0 status

The principles of user-centered design were applied to develop an interactive web interface and provide a decision support tool to predict the patient’s probability of healthy axillary lymph nodes. Specifically, the guiding principles of risk communication (27, 28) have been implemented in the web-based format of the NILS tool and involve the following:

I) Presenting the numeric estimate of the probability of N0 for an individual patient.
II) The estimate was interpreted in the context of the background distribution of the estimated probabilities of healthy lymph nodes in the breast cancer population used for model development.
III) Conveying uncertainties and disclaimers related to current research status.
IV) Combining numerics with a visual display to aid comprehension.
V) Tailoring estimates with a personalized message for each individual output.

The design was developed in collaboration with Medos AB, an external medical software company (https://www.medos.se). Graphical features allow users to set up the NILS interface on computers and common mobile devices, such as smartphones and tablets.

### 2.6 Technical validation of the NILS interface

To validate that the implementation of the NILS tool followed the design specifications, the NILS interface (version 0.1.0) was validated with 100 reference test subjects, selected to cover different combinations of missing histopathological input data. The reference dataset was based on the development cohort, in which the following random patients were selected:

- 25 patients with a complete dataset.
- 25 patients with a complete dataset, excluding VI.
- 25 patients in whom the ER status, PR status, or Ki67 value is missing.
- 25 patients where VI is missing in conjunction with a missing value in at least one of ER status, PR status, or Ki67 value.

Clinicopathological data were assigned for manual input into the interface. The NILS tool calculated a prediction value and identified a cut-point for each datapoint in the reference dataset. The exact value from the NILS tool was obtained from web browser console output, since the value displayed in the graphical user interface is rounded to integer value. The results of the predictions made through NILS was compared to the results obtained through the reference ANN algorithms.

## 3 Results

### 3.1 Clinical and histological characteristics

Data from a consecutive cohort of 800 invasive breast cancer cases, one observation per case, were included in the training and internal validation of the original ANN prototype for healthy lymph nodes. The benign axillary nodal status was displayed in 516 (64.5%) cases. Patients with N0 breast cancer had smaller tumors and a higher rate of mammography-detected disease than those with N+ status (**Supplemental Table 1**). Furthermore, breast tumors of patients with N0 displayed a higher proportion of ER negative status, had lower levels of Ki67, and were more often lacking multifocality and VI.

### 3.2 Model performance

#### 3.2.1 Predictive performance and calibration for the estimation of VI

**Figure 3:**
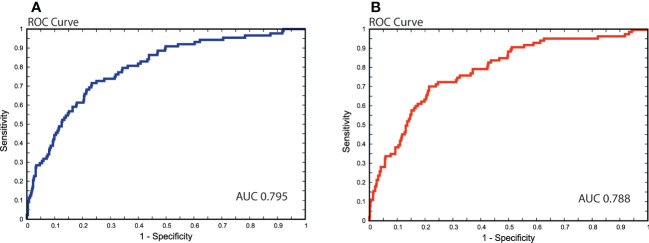
Receiver operating characteristics (ROC) curves visualizing predictive performance for estimating vascular invasion. Area under the curve (AUC) **(A)** Model comprising of complete input data (9 variables); **(B)** Model without the three input variables: estrogen receptor (ER), progesterone receptor (PR), and Ki67.

In cases with missing data on VI, one of two separate ANN models was chosen to acquire an imputed value for VI. The model for VI imputation comprising complete input data (9 variables) achieved an AUC of 0.795 (95% confidence interval [CI], 0.751−0.835), **Figure 3**. If any of the input variables, ER status, PR status, or Ki67 value were missing, an ANN model without these inputs was applied for VI prediction and displayed a discriminatory performance of AUC 0.788 (95% CI, 0.744−0.831). The corresponding median 8-degree of freedom (df) HL statistic values were 7.5 (*P* = 0.480) and 13.4 (*P* = 0.100), respectively.

#### 3.2.2 Predictive performance and calibration for the prediction of N0

**Figure 4:**
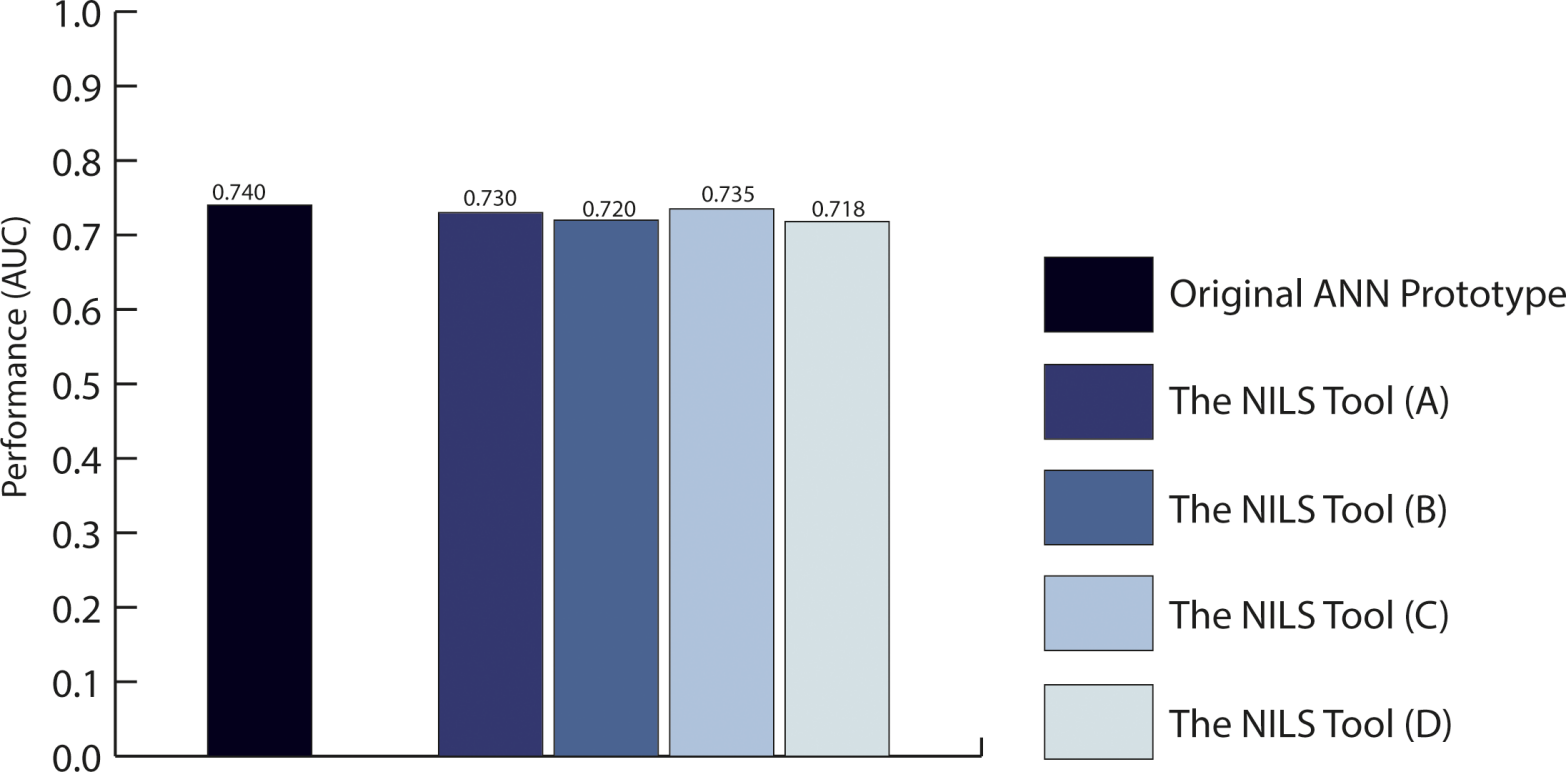
Predictive performance to discriminate healthy lymph node status at the time of breast cancer diagnosis. The diagram displays the area under the receiver operating characteristics curves (AUCs) for the original ANN prototype presented in 2019, which required 15 input variables and the ANN pathways within the present NILS tool, which requires the maximum number of 10 top-ranked input variables. **(A)** The NILS tool that includes all top-ranked variables and random imputation of missing data; **(B)** The NILS tool with top-ranked variables excluding estrogen receptor (ER), progesterone receptor (PR), and random imputation of missing data; **(C)** The NILS tool that includes all top-ranked variables, vascular invasion (VI) is estimated if missing, random imputation of the remaining missing data; **(D)** The NILS tool with top-ranked variables excluding ER, PR, and Ki67. VI is estimated if missing random imputation of the remaining missing data.

The present NILS prediction tool to distinguish healthy axillary lymph nodes with complete data and, if necessary, the prediction of VI displayed good discrimination with a calculated AUC of 0.735 (95% CI, 0.704−0.764). The NILS tool was well calibrated, with an observed 8-df-HL statistic of 9.9 *(P* = 0.270). The prediction model without input values for ER status, PR status, and Ki67 achieved a discriminatory performance of AUC 0.718 (95% CI, 0.687−0.748). The corresponding median 8-df-HL statistic value for this model was 9.7 (*P* = 0.290). **Figure 4** shows the discriminative ability (N0 vs. N+) of different ANN pathways within the NILS tool.

#### 3.2.3 Performance of the NILS tool compared to the previous ANN prototype for N0

The initial ANN prototype presented in 2019 to distinguish healthy lymph nodes (N0 vs. N+), comprising 15 potential clinicopathological predictors, achieved a mean validation AUC of 0.735. The corresponding internally validated AUC for N0 was 0.740 (95% CI, 0.723–0.758) (23). Compared with the present NILS tool, which includes a reduced number of input variables and allows missing values for VI, ER, PR, and Ki67, there was a 0.022-point estimate difference between the AUCs, as shown in **Figure 4**.

### 3.3 Implications for sentinel lymph node reduction rate

**Table 1** shows the possible SLNB reduction rates after applying the NILS tool to assign healthy lymph nodes (N0 vs. N+) and accepting missing values for VI, ER, PR, and Ki67. A SLNB reduction rate of 26% would be attained by applying the present NILS tool at a cut-off point corresponding FNR of 10% to discriminate N0. At this cut-off point, sensitivity and specificity values of 90% and 34%, respectively, were demonstrated.

**Table 1:** Possible sentinel lymph node biopsy (SLNB) reduction rates at cut-point equivalent to the maximum false negative (FNR) rate of 10%, reflecting the accepted FNR of the SLNB procedure.

Model (N0 versus N+)	TP	TN	FP	FN	Sensitivity	Specificity	FNR	SLNB reduction rate
ANN Prototype*	258	190	324	28	91%	37%	<10%	27%
**The NILS Tool**	256	177	339	28	90%	34%	<10%	26%

N0, No axillary lymph node involvement (benign lymph nodes); N+, axillary lymph node positive (metastatic lymph nodes); TP, true positive; TN, true negative; FP, false positive; FN, false negative; FNR, false negative rate; SLNB, sentinel lymph node biopsy.

*Dihge L, Ohlsson M, Eden P, Bendahl PO, Ryden L. Artificial Neural Network Models to Predict Nodal Status in Clinically Node-Negative Breast Cancer. BMC cancer 2019 (23).

Comparison between potential SLNB reduction rates by applying the former ANN prototype (15 input variables)* and the current NILS tool (maximum 10 input variables) trained to tolerate missing preoperative input values for vascular invasion, estrogen receptor (ER), progesterone receptor (PR), Ki67.

### 3.4 The NILS interactive decision support tool

#### 3.4.1 Background distribution curves

**Figure 5:**
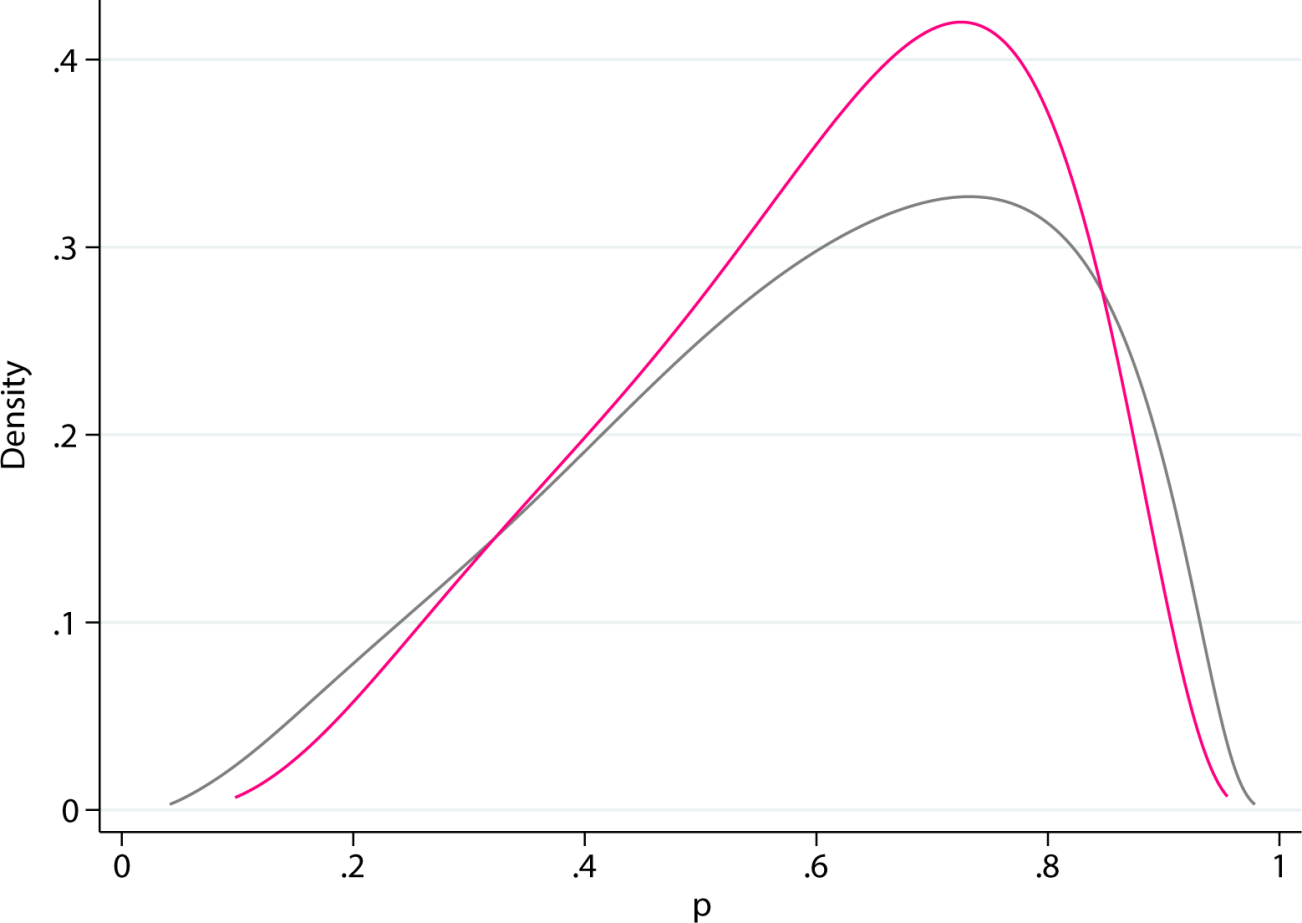
Smoothed background distributions of the predicted probabilities (p). Gray: all input values present. Pink: missing estrogen receptor (ER), progesterone receptor (PR), and Ki67.

The workflow for the prediction of N0 probability consisted of the ANN model with complete data for all input variables, including a possible imputation of VI, and the ANN model with missing values for any of ER, PR, and Ki67. For each model, the output was the predicted probability (*p*) of healthy lymph nodes (N0). The smoothed empirical distribution of the predicted probabilities obtained during model development is shown in **Figure 5**. The smoothing procedure includes a transformation of the empirical probabilities to logits, that is, ln(*p*/(1-*p*)), where the predicted probability *p* is a value between 0 and 1, and thereafter kernel smoothing using the command k-density in the statistics package Stata/MP 17.0. A Gaussian kernel with a bandwidth of 0.5 was used. The logit transformation ensures that the support of the distribution remains within the interval 0 < *p* < 1.

#### 3.4.2 The interactive web-based NILS interface

**Figure 6:**
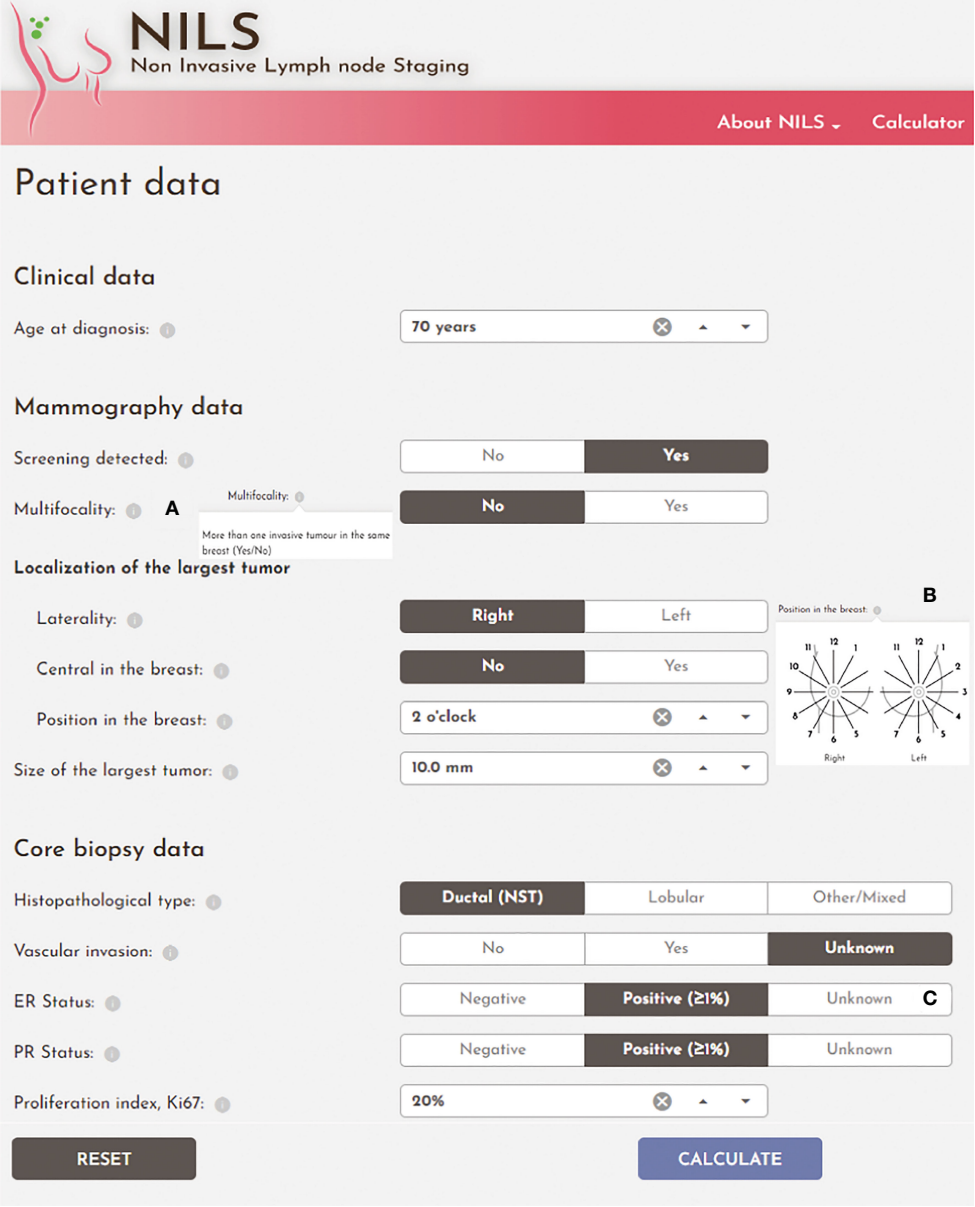
The input screen of the online NILS interface. Clinicopathological and mammographical input entries for the prediction of healthy lymph nodes with essential user interface elements: **(A)** Clickable information icons with explanatory information text; **(B)** Clarifying illustration of the required input entry; **(C)** Option to choose “Unknown” or “Undefined” icons for input variables: vascular invasion, estrogen receptor (ER), progesterone receptor (PR), and Ki67.

**Figure 7:**
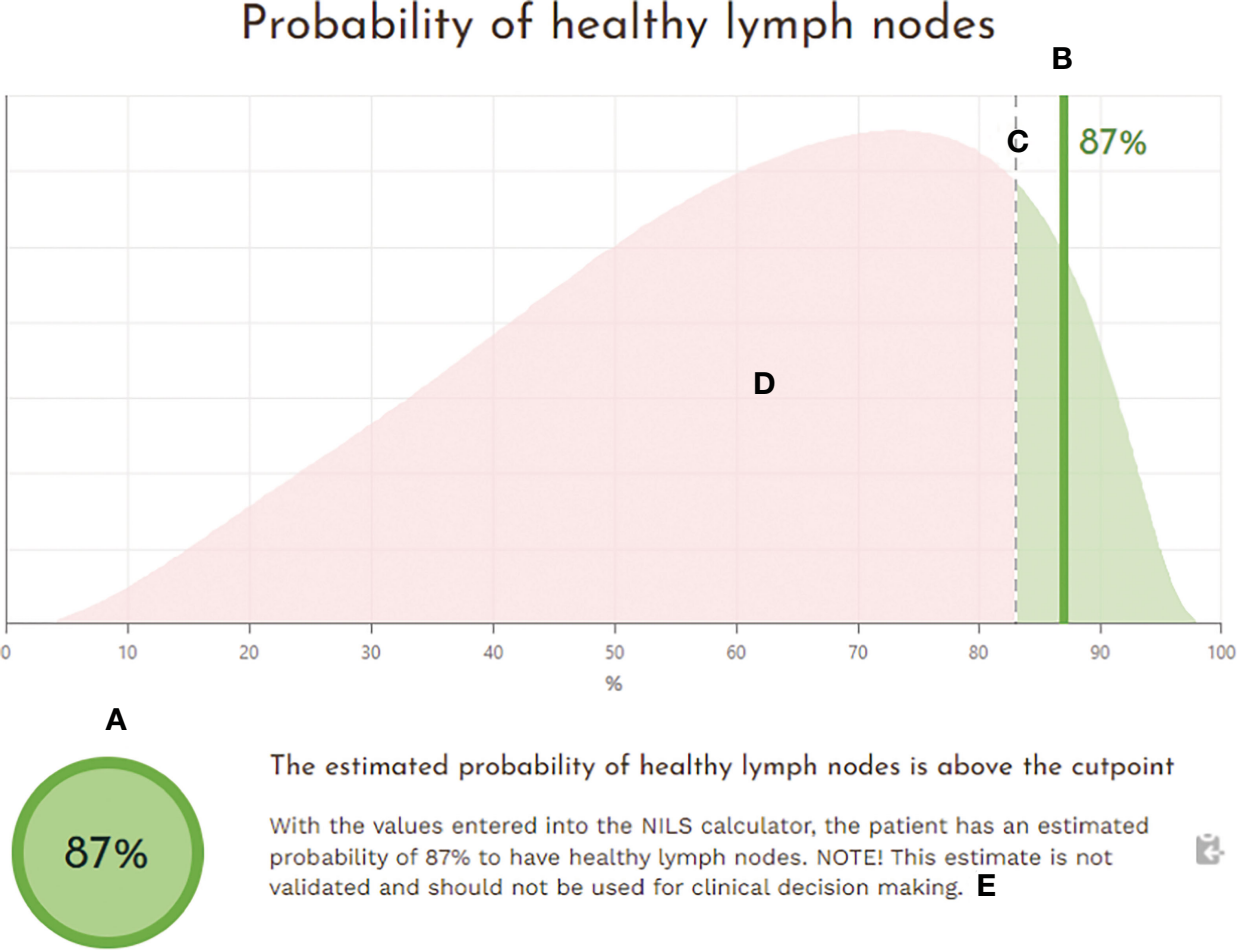
The output field of the NILS interface with an estimated probability of healthy lymph nodes at the time of breast cancer diagnosis. The output is presented **(A)** numerically as an estimated probability of healthy lymph nodes for the individual patient and **(B)** graphically as a vertical line in relation to **(C)** the set cut-off point for a binary outcome (N0 vs. N+). The graphical output is illustrated superimposed on **(D)** a smoothed histogram describing the distribution of estimated probabilities for healthy lymph nodes in the population used for model development and **(E)** a tailored text message conveying the uncertainty of the prediction related to current research status.

The ANN algorithms for nodal status prediction have been implemented into a fully operative web-based decision support tool. The NILS interactive prediction tool for healthy lymph nodes was built to incorporate features of a user-centered design. Using 10 entry values with information on the patient’s age, data from mammography examination, and core needle biopsies from the breast tumor, the tool can predict the likelihood of having healthy lymph nodes at the time of breast cancer diagnosis. The NILS interface includes clickable information icons that display explanations of the input fields, as shown in **Figure 6**. The ANN algorithms of the NILS tool predict the probability of healthy lymph nodes, which are displayed graphically together with the applicable cut-off point. Therefore, the outputs are presented numerically, as the patient’s individual estimated probability of healthy lymph nodes and graphically in relation to the set cut-off point for the binary outcome (N0 vs. N+). Individual estimation is visualized graphically as a vertical line superimposed on a smoothed histogram, which describes the distribution of estimated probabilities for healthy lymph nodes in the population used to develop the NILS tool. In addition, the suggested cut-off point is superimposed on this distribution. **Figure 7** shows an output display that incorporates these features. The output score was compared to the specified cut-off point for binary classification (N0 vs. N+). The result of this comparison is positive if the prediction is above the cut-off point (the green icon assigns a probability of N0 above the cut-off point). If the prediction is below the cut-off point, the result is negative (the red icon assigns a probability of N0 below the cut-off point).

The NILS tool was submitted to an initial usability test to assess the clinical functionality of the interface and to identify necessary alterations to the layout and interactive elements, such as input and output fields, information icons, and explanatory information text. Each individual estimate of nodal status prediction includes a disclaimer to communicate the uncertainty related to the current research status. The NILS website has been translated into English and can be accessed at https://nils.thep.lu.se/.

To ensure quality assurance for future clinical preoperative applicability, the NILS predictive tool is now applied in a clinical trial for validation purposes without interfering with the current breast cancer workflow (ISRCTN registry, study ID 14341750) (29). The prediction tool or “calculator” is currently accessible within the research program by entering login credentials.

#### 3.4.3 User interface validation

NILS version 0.1.0 was tested with a total of 100 reference test patients selected to cover combinations of missing histopathological input data. Two subjects with no information on multifocality were mistakenly included (did not fulfilled the eligibility criteria for the validation) and generated incorrect test results. For the rest, 98 tested subjects, the estimated probability of healthy lymph nodes made using the NILS tool matched the predictions obtained through the reference algorithm to the 8^th^ decimal. The cut-point and graphical curve presented on the output screen were correct for all test subjects.

## 4 Discussion

In this study, we present an implementation of ANN algorithms for the prediction of healthy lymph nodes as a decision support tool for a personalized, noninvasive lymph node staging in breast cancer. The NILS tool is the first interactive tool based on ANN to complement the updated ASCO guideline, which recommend abstaining SLNB in selected patients on a case-by-case basis (17). The NILS tool allowed missing histopathological input values, and achieved predictive performance with an AUC ranging from 0.718 to 0.735 with good calibration. By applying the NILS tool for nodal status and setting the cut-off point for model classification to reflect the accepted FNR of the SLNB procedure, the potential to reduce surgical axillary intervention by 26% was demonstrated.

The clinical utility of the NILS tool for noninvasive prediction of healthy lymph nodes depends on the information obtained in the preoperative setting. To handle the risk of incomplete preoperative histological reporting based on core needle biopsy (CNB), the ANN algorithm in the NILS tool incorporated strategies to allow missing data for VI and/or ER status, PR status, and Ki67 value. Previous studies have shown high specificity but low sensitivity for VI detection in CNB (30) with a reported 30% detection failure rate (31). Therefore, a separate model for estimating VI is integrated into the present NILS tool. CNB samples have been shown to be accurate in determining the ER and PR status (32), however, a significant difference between Ki67 values in CNB and surgical specimens has been observed, with a relative decrease from the biopsy to surgical sample (33). As high Ki67 expression has been associated with axillary nodal metastasis (34), the NILS tool may overestimate the risk of axillary nodal spread in the preoperative setting, indicating appropriate cautiousness of the tool.

Previously, models have been developed to predict sentinel lymph node status using a variety of clinical and pathological factors (35, 36). One of the first was the Memorial Sloan-Kettering Cancer Center nomogram based on logistic regression analysis to estimate the probability of SLNB metastasis, which provided an AUC of 0.754. The final variable selection in the presented NILS tool focused on clinical knowledge, previous literature and variables associated with nodal status in the present cohort. The ten highest-ranking variables were included: tumor size, vascular invasion, multifocality, ER status, histological type, PR status, mode of detection, age, tumor localization, and Ki67 all of which have repeatedly been reported to be of value for predicting nodal status in breast cancer. Machine learning-based algorithms for predicting axillary nodal spread have been proposed to reflect the complex multifactorial process of lymph node metastasis (37, 38). Especially, artificial intelligence with a deep learning approach in radiological image recognition, has gained attention to predict axillary nodal metastasis (39, 40). However, the clinical significance of these findings in routine preoperative settings remains limited. ANNs are usually included in the group of machine learning techniques described as black-box models and are often criticized as nontransparent. For a given output, it is typically difficult to gain a comprehensive understanding of the relative importance of each input variable (41). Visualization techniques based on sensitivity analysis have been proposed (42). Consequently, to better comprehend the significance of each input variable in the ANN algorithm within the NILS tool, sensitivity analysis was previously applied to the variable selection process. In the publication from 2019, mean odds ratios was presented with the corresponding percentiles to highlight the dynamic nature of the 10 highest-ranking input variables for the prediction of nodal status (23).

Digital clinical decision support systems in breast cancer surgical care remains sparse (43). Diverse predictive methods has been applied on the benefit of adjuvant chemotherapy in the different systems, such as OncoType DX^®^ embedded in a decision support algorithm (44) and cumulative hazard function by PREDICT (45). Hitherto, diagnostic decision support tools have not had as much impact as other types of clinical decision support systems (46). Reasons identified included negative perceptions in health care, inferior accuracy due to missing input data and poor integration of the tools with medical records (46, 47). Although the overall performance of the NILS tool was moderate (AUC 0.720-0.735), the tool displayed a high sensitivity (90%) to distinguish breast cancer patients most likely to harbor healthy lymph nodes. The ANN algorithms also allow different cut-off points to be assigned, which can be determined by the actual clinical setting. The reduction from the mandatory 15 input variables previously required (23) to 10 variables in the updated NILS tool, with an allowance of missing input entries with only 0.005−0.022 decrease in AUC point estimate, strengthens the clinical utility and reinforces the potential to spare one in four patients the surgical SLNB procedure.

Given the increasing evidence around de-escalating axillary surgery, the Choosing Wisely statement (48) declared that SLNB is not required for women ≥ 70 years with clinically node-negative (cN0) breast cancer, which is hormone receptor-positive and HER2-negative if they are adjuvantly treated with hormonal therapy. Other advocates omitted SLNB in all low-risk patients aged ≥75 years, even without planned hormonal treatment (49). Randomized trials, such as SOUND (50) and INSEMA (51), now address the possibility of omitting SLNB and include patients of all ages with ultrasonographically disease-free axillae. Although advances are promising, the sensitivity of routine axillary ultrasound alone is considered insufficient to replace SLNB (52). The updated ASCO guideline on the management of the lymph nodes in early breast cancer state that patients should be evaluated on a case-by-case basis to ensure patient-centered care (17). Consequently, decision support tools that consider the patient’s individual preoperative clinicopathological variables for nodal status prediction could contribute to a better differentiation of breast cancer patients with disease-free axilla from those with nodal metastasis.

An important step before clinical implementation of a decision-support tool is to consider both associated health care costs and impact on patients´ quality of life under plausible scenarios. Compared to the standard of care with SLNB, the adoption of the NILS tool for noninvasive staging of nodal status has been shown to promote reduced health care costs and gains in quality of life, especially in patients undergoing breast-conserving surgery (24). Although the NILS prediction tool has been developed for clinical use following the framework of the Tripod checklist (53) to increase the methodological consistency and quality of the prediction models, the true validity of the model should be assessed in a fully independent dataset. To address this, a validation study is currently being conducted based on two external cohorts that assess the geographic and temporal validity of the NILS prediction tool (ISRCTN 14341750) (29).

Limitations of the NILS tool include that the model was developed using data mostly from patients with screen-detected breast cancer (57%, **Supplemental Table 1**). This is because Sweden has a population-based mammography screening program with an overall attendance of >80% (54). The NILS tool is not designed for settings with limited access to population-based screening, but the current tool can provide a range of probabilities of healthy lymph nodes in patients with screen-detected *versus* symptom-detected breast cancers. Detection by mammography screening appears to be an independent factor associated with lower risk of axillary lymph node involvement (55). Another limitation is that variation in the proportions of node-positive breast cancer may also produce errors in the accuracy of the tool. However, procedures to adjust outputs of a classifier to new *a priori* probability has been proposed to mitigate these errors and might be applied in the future (56). A third limitation is that no differentiation was made between micrometastases and macrometastases using the current definition of nodal metastatic lesions (N+). While this may demonstrate the appropriate cautiousness of the NILS tool by appointing the classification task to differentiate healthy lymph node status from those harboring any metastasis, the finding of only micrometastatic deposits in sentinel nodes has no longer relevance as a criterion for completion of axillary surgery (8, 57). Similarly, the decision on adjuvant systemic therapy is mainly based on tumor biomarkers and/or gene profiling rather than on identifying low-burden nodal metastasis (58). However, the presence of nodal macrometastasis in breast cancer remains relevant for decision-making regarding adjuvant radiotherapy (59). Ongoing research efforts to overcome this limitation include validating the NILS tool in datasets that specify the distinction between micrometastasis and macrometastasis in sentinel lymph nodes. Improving efficacy of systemic treatment regimens in breast cancer can pave the way to further expand de-escalation of axillary surgery and safe omission of SLNB for more patients. Thus, the NILS tool needs to be flexible to new target populations and adapt accordingly. The validity of the NILS tool will be assessed in patients undergoing different breast surgical approaches (breast-conserving surgery vs. mastectomy). Furthermore, the predictive performance of the NILS tool with additional features will be evaluated, including the incorporation of mammography-based radiomics model for the prediction of axillary lymph node status.

## 5 Conclusion

This study reports the implementation of ANN algorithms for axillary nodal status prediction as a web-based decision supporting tool for noninvasive lymph node staging. The NILS tool uses 10 routinely available entry values and allows missing histopathological data on core needle biopsies to predict the likelihood of benign lymph nodes at the time of breast cancer diagnosis. The NILS tool is a personalized decision support tool based on ANN to complement the updated ASCO guideline, which recommends refraining from SLNB in selected low-risk patients after evaluation on a case-by-case basis. By applying the NILS tool for preoperative prediction of lymph node status, the potential to avoid surgical axillary staging was demonstrated in about one in four patients. In addition, we have shown substantial cost reductions and overall health gains associated with the implementation of the NILS tool. This study highlights the potential of ANN-based prediction tools in clinical use to aid in diagnosis and reduce unnecessary axillary surgery in early breast cancer.

## Data availability statement

The data analyzed in this study is subject to the following licenses/restrictions: The datasets generated and/or analyzed during the current study are not publicly available because of privacy and ethical restrictions but are available from the corresponding authors (LD or LR) on reasonable request. Requests to access these datasets should be directed to LD, looket.dihge@med.lu.se or LR, lisa.ryden@med.lu.se.

## Ethics statement

This study was approved by the Ethics Committee of Lund University (LU 2012/340). The study received ethical approval for the use of an opt-out methodology and written informed consent for participation was not required for this non-interventional study in accordance with the national legislation and the institutional requirements.

## Author contributions

Conceptualization, LR, MO, and LD; methodology, LR, LD, MO, and P-OB.; software, MO; validation, MO; formal analysis, MO, LD, and P-OB; investigation, LD, LR; resources, LR and MO; data curation, LD, MO, and P-OB; writing – original draft preparation, LD; writing – review and editing, MO, LR, P-OB, IS, and MH; visualization, LD, LR, and P-OB; supervision, LR and MO; project administration, LR; funding acquisition, LR. All authors contributed to the article and approved the submitted version.
