# Supplementary material for: The implementation of NILS: A web-based artificial neural network decision support tool for noninvasive lymph node staging in breast cancer
Source: Front Oncol. 2023 Mar 1;13:1102254. doi: 10.3389/fonc.2023.1102254 (PMC10014909; doi:10.3389/fonc.2023.1102254)
Supplement: Supplementary file 1 [file Table_1.docx]

**Supplemental Table 1.** Patient and tumor characteristics.

|  | All (*n*=800) | N0 (*n*=516) | N+ (*n*=284) | *P* value^‡^ |
| --- | --- | --- | --- | --- |
| Age^a^, years  Missing | 64 (24-92)  0 | 66 (33-91)  0 | 64 (24-92)  0 | 0.050^§^ |
| Mode of detection  Mammographic screening  Symptomatic presentation  Missing | 457 (57)  343 (43)  0 | 323 (63)  193 (37)  0 | 134 (47)  150 (53)  0 | <0.001 |
| Multifocality  Absent  Present  Missing | 610 (77)  179 (23)  11 | 419 (82)  93 (18)  4 | 191 (69)  86 (31)  7 | <0.001 |
| Tumor site with the breast  Central  Upper inner quadrant  Lower inner quadrant  Upper outer quadrant  Lower outer quadrant  Overlapping lesions: 3, 6, 9, 12 o’clock  Missing | 22 (3)  108 (14)  46 (6)  266 (33)  84 (11)  274 (34)  0 | 14 (3)  78 (15)  32 (6)  167 (32)  43 (8)  182 (35)  0 | 8 (3)  30 (11)  14 (5)  99 (35)  41 (14)  92 (32)  0 | 0.059 |
| Tumor size^a^, mm  Missing | 15 (0.5-90)  1 | 13 (0.5-70)  0 | 18 (0.9-90)  1 | <0.001^§^ |
| Histological type  NST / Ductal  Lobular  Other  Missing | 640 (80)  101 (13)  59 (7)  0 | 410 (80)  60 (12)  46 (9)  0 | 230 (81)  41 (14)  13 (5)  0 | 0.052 |
| Vascular invasion  Absent  Present  Missing | 545 (85)  94 (15)  161 | 403 (94)  28 (7)  85 | 142 (68)  66 (32)  76 | <0.001 |
| ER status  Positive (≥1%)  Negative (<1%)  Missing | 729 (91)  69 (9)  2 | 461 (90)  53 (10)  2 | 268 (94)  16 (6)  0 | 0.025 |
| PR status  Positive (≥1%)  Negative (<1%)  Missing |  |  |  | 0.019 |
|  | 673 (84)  125 (16)  2 | 422 (82)  92 (18)  2 | 251 (88)  33 (12)  0 |  |
| HER2 status  Positive  Negative  Missing | 86 (12)  647 (88) | 52 (11)  423 (89) | 34 (13)  224 (87) | 0.370 |
|  | 67 | 41 | 26 |  |
| Ki67^a^, %  Missing | 15 (0-94) | 14 (0-94) | 17 (1-81) | 0.001^§^ |
|  | 48 | 29 | 19 |  |

Note: the *P*-values are unadjusted and refer to comparisons between N0 and N+. Column percentages are given for categorical variables unless indicated otherwise. The percentage has been rounded and, therefore, may not sum to 100. ^‡^ Pearson’s χ^2^ test with degrees of freedom equal to the number of categories minus one, except for ^§^Mann–Whitney U test, ^a^ median (range).
N0, no axillary lymph node involvement (benign lymph nodes); N+, axillary lymph node-positive (metastatic lymph nodes); NST, invasive carcinoma of no special type; ER, estrogen receptor; PR, progesterone receptor.
